# Single-nucleus RNA-sequencing reveals the cellular programs driving nematode-induced giant cell formation in tomato

**DOI:** 10.1093/hr/uhaf223

**Published:** 2025-08-22

**Authors:** Sobhan Bahrami Zadegan, Peitong Li, Mst Shamira Sultana, Hafiz Muhammad Khalid Abbas, Nicole Coffey, Cengizhan Öztürk, Mariam Elwasif, John Hollis Rice, Hari B Krishnan, Tarek Hewezi

**Affiliations:** Department of Plant Sciences, University of Tennessee, Knoxville, TN 37996, USA; UT-ORNL Graduate School of Genome Science and Technology, University of Tennessee, Knoxville, TN 37996, USA; Department of Plant Sciences, University of Tennessee, Knoxville, TN 37996, USA; Department of Plant Sciences, University of Tennessee, Knoxville, TN 37996, USA; Department of Plant Sciences, University of Tennessee, Knoxville, TN 37996, USA; Department of Plant Sciences, University of Tennessee, Knoxville, TN 37996, USA; Department of Plant Sciences, University of Tennessee, Knoxville, TN 37996, USA; UT-ORNL Graduate School of Genome Science and Technology, University of Tennessee, Knoxville, TN 37996, USA; Department of Plant Sciences, University of Tennessee, Knoxville, TN 37996, USA; Department of Plant Sciences, University of Tennessee, Knoxville, TN 37996, USA; Plant Science Division, University of Missouri, Columbia, MO, USA; Plant Genetics Research, The United States Department of Agriculture (USDA) Agricultural Research Service, Columbia, MO, USA; Division of Plant Science and Technology, University of Missouri, Columbia, MO, USA; Department of Plant Sciences, University of Tennessee, Knoxville, TN 37996, USA

## Abstract

Plant-parasitic root-knot nematodes (*Meloidogyne* species) are highly polyphagous parasites that alter cellular identity of terminally differentiated root cells to induce the formation of giant cells and knot-like structures known as galls, whose ontogeny remains largely unknown. In this study, we generated single-nucleus RNA-seq data of galls and neighboring root tissues at two distinct stages of *Meloidogyne incognita* infection of tomato (*Solanum lycopersicum*) plants. Analysis of 35 393 high-quality nuclei resulted in the identification of three stele-associated cell clusters that captured young and more differentiated giant cells, where 772 genes were preferentially expressed. Giant cell-specific expression patterns of a set of these genes were validated using promoter activity assays. We used pseudotime analysis to trace how gene activity changes as giant cells develop. Developmental trajectory analysis revealed a gradual activation of more complex gene regulatory networks as young giant cells adopt specific fates and become more differentiated. Functional assays using gene silencing confirmed the functional importance of giant cell-expressed genes in mediating plant susceptibility to *M. incognita*. Cell type-specific gene expression analysis revealed that xylem, phloem, stele, endodermal, and protophloem cells undergo extensive transcriptome reprograming, which facilitates coordinated cellular responses to nematode infection, including immune signaling, structural support, and metabolic adjustments. Together, our analyses represent the first single-nucleus transcriptomic map of nematode-induced giant cells and provide novel insights into the molecular events leading to the formation of a new plant organ and feeding cells orchestrated by an animal parasite.

## Introduction

Root knot nematodes (*Meloidogyne* sps.) are sedentary endoparasites of root systems of >2000 plant species, including many of economic importance [1]. During the compatible interaction, the infective second-stage juveniles (J2s) redifferentiate terminally differentiated vascular cells into enlarged feeding cells, known as giant cells. The formation of giant cells, typically between four and nine, also stimulates surrounding cells to divide and enlarge in size, resulting in the formation of knot-like structures known as galls [2, 3]. The formation of giant cells by root-knot nematodes is a fascinating example of how an animal can manipulate plant cellular differentiation and developmental pathways to its advantage. This process demonstrates a sophisticated form of cross-kingdom interactions, where an animal induces specific changes in plant cells, transforming them into highly specialized structures that support its survival and reproduction.

Recent research has provided insight into the molecular mechanisms underlying the ontogeny of giant cells as specialized feeding cells. Upon infection, infective juveniles induce massive transcriptome programming of target cells that includes upregulation of genes associated with cell cycle activation, cell wall modifications, cytoskeleton remodeling, and metabolic pathways while suppressing defense- and immunity-related genes [4–7]. Transcriptome reprogramming of infected root cells activates endoreduplication process, resulting in enlargement of nuclei and increased cellular DNA content, a key characteristic of metabolically hyperactive cells [8]. Transcriptional regulation of genes related to cytoskeleton rearrangement and cell wall modifications facilitates the enlargement of giant cells and accommodates the proliferation of organelles such as mitochondria, plastids, and vacuoles [3, 8]. Transcriptome analysis also supports the biosynthesis of essential metabolites and transport of nutrients into giant cells, ensuring a steady food supply for the nematode throughout the parasitic interaction [3–8].

The regulation of diverse gene networks in a spatially and temporally coordinated manner during gall and giant cell formation appears to involve the activity of a large number of transcription factors of various gene families [7, 9]. DNA methylation, histone modifications, and accumulation of small and long noncoding RNAs have also recently emerged as functional regulators of gene expression during nematode parasitism of host plants [10–16]. This may add significant complexity to gene expression regulation during nematode infection but also ensure flexibility of gene activation and deactivation at different parasitic stages, enabling plants to adapt to the nematode-induced reprogramming.

Determining transcriptome signatures of giant cells is critical for understanding the mechanisms through which plant-parasitic root-knot nematodes induce cellular reprogramming and transform root cells into specialized feeding cells. However, the resolution and specificity of transcriptome rewiring of giant cells are limited by using whole-root or gall tissues in which giant cells constitute only a very small fraction. In addition, giant cells are embedded within complex tissues, surrounded by other cell types with distinct transcriptional profiles, leading to challenges in separating their unique expression patterns. To enhance resolution and specificity in transcriptomic profiling, laser microdissection has been used to isolate giant cells from various plant species [17–21]. However, this approach is limited by its labor-intensive nature, susceptibility to RNA degradation, and lack of single-cell resolution and high throughput, ultimately constraining its ability to capture cellular heterogeneity and detect rare cell types. In this context, recent advances of single-cell RNA-sequencing (scRNA-seq) have facilitated the identification of transcriptome signatures of various plant cell types at unprecedented single-cell resolution [22–28]. Considering the large size and irregular shape of giant cells, single-nucleus RNA-seq (snRNA-seq) is ideally suited to determine the transcriptome of nematode-induced giant cells because nuclei can be isolated without harsh enzymatic or mechanical treatments, yielding accurate nuclear transcriptomes with significantly fewer dissociation-induced stress and size-based capture biases than whole-cell methods.

In this study, we generated snRNA-seq data of galls and neighboring root tissues at two distinct stages of *Meloidogyne incognita* infection of tomato plants. We identified cell clusters that captured young and fully developed giant cells. Specifically expressed genes in these clusters were sorted by pseudotime, allowing for the prediction of giant cell developmental trajectories. We identified gene activation patterns and the regulatory mechanisms underlying temporal progression of giant cell differentiation. We also identified nematode-responsive genes in xylem, phloem, stele, endodermis, and protophloem, providing the first insights into how plants detect and respond to nematode invasion at the cellular level. Furthermore, the specificity and functional importance of giant cell preferentially expressed genes were experimentally validated.

## Results

### Generation of snRNA-seq libraries of galls and neighboring root cells

**Figure 1:**
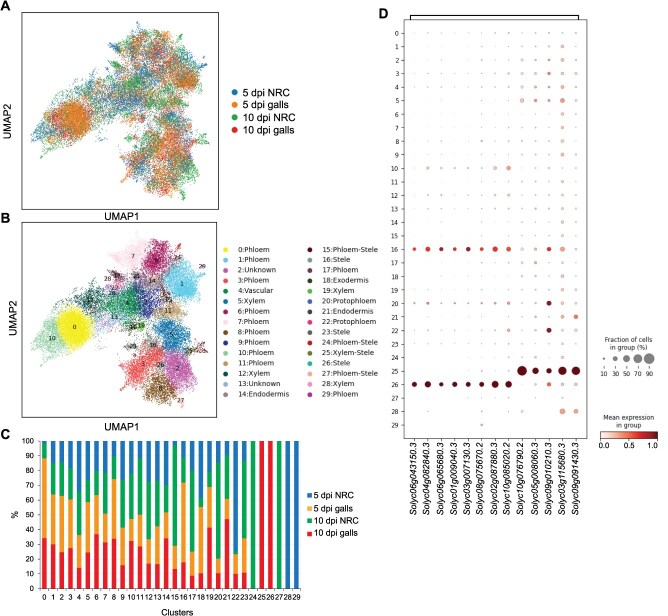
Single-nucleus transcriptome atlas of galls induced by *M. incognita* in tomato. (a, b) UMAP plots of galls and NRC collected at 5 and 10 days post-*M. incognita* infection. Nuclei are colored based on tissue identities (a) or assigned clusters and cell types (b). (c) Stacked bar graph depicting the proportion of nuclei from each of the four snRNA-seq libraries across all 30 clusters. (d) Dot plot showing the expression of 13 giant cell marker genes across all clusters. Dot diameter indicates the percentage of cells expressing a given gene in the indicated cluster. The color bar on the right indicates relative gene expression.

We generated single-nucleus transcriptome of *M. incognita*-induced galls on tomato roots at 5 and 10 days postinfection (dpi), which represent early- and fully developed galls. At the same infection stages, ~1 cm of gall-neighboring root tissues were also collected and used as control as previously described [7]. Neighboring tissues share similar developmental stages and cell type compositions with the gall region prior to infection. Thus, using gall neighboring root cells (NRC) as a control reduces biological variability and batch effects introduced by using noninfected whole roots or root segments from different zones and developmental stages, thereby improving the sensitivity and accuracy of differential gene expression and cell type identification analyses. After data processing and filtering, a total of 35 393 high-quality nuclei were analyzed, including 6302 nuclei from 5-dpi galls, 11 140 from 5-dpi NRC, 8274 from 10-dpi galls, and 9677 from 10-dpi NRC (Table S1). A median number of 690 expressed genes per nuclei was determined (Table S1). After normalization and scaling of gene expression levels, nuclei from both galls and NRC were clustered and visualized using uniform manifold approximation projection (UMAP) method (Fig. 1a). This resulted in the identification of 30 distinct cell clusters (Fig. 1b). Previously validated and conserved orthologs of cell type-specific marker genes (Table S2, Fig. S1) were then used to assign each cluster to a specific cell type (Fig. 1b).

### Identification of giant cell specifically expressed genes

Our analysis revealed overlaps between gall and NRC samples as well as between the two time points except for clusters 24, 25, 26, 27, 28, and 29 (Fig. 1c and Table S3). Clusters 28 and 29 contained cells unique to the 5-dpi NRC, whereas cluster 24 and 27 contained cells unique to the 10-dpi NRC (Fig. 1c and Table S3). Interestingly, clusters 25 and 26 contained cells exclusive to the 10-dpi galls. Cluster 25 contained 126 cells and was annotated as xylem cells. Similarly, cluster 26 contained 125 cells and was annotated as stele cells (Fig. 1c and Table S3). The presence of distinct clusters for xylem (5, 12, 19, and 28), phloem (0, 1, 3, 6–11, 17, and 29), stele (16, 23, and 26), endodermis (14 and 21), and protophloem (20 and 22) implies that there were cells within each of these cell types whose transcriptome are different. Clusters 2 and 13 were annotated as ‘unknown’ because they lack clear, cell type-specific marker expression and may represent novel, transitional, or rare cell states.

Considering that *M. incognita* induces the formation of giant cells in the vascular tissues, we examined the possibility that clusters annotated as vascular, stele, or xylem captured nematode-induced giant cells. Thus, the expression of several genes previously shown to be expressed during early or late stages of giant cell formation were examined in all clusters. These genes included the *CELL CYCLE SWITCH PROTEIN 52* (*CCS52B*), the *cyclin-dependent kinases cdc2b*, *CycB1;1* and *CycA2;1*, the *65-kDa microtubule-associated protein MAP65–3* (*MAP65–3*), *alpha- and beta-tubulins*, and *Formin 2B*, which have been shown to be expressed in giant cells during early stages of infection [29–33]. Other marker genes that have been shown to be expressed in giant cells during late stage of infection include *auxin resistant 1* (*Aux1*), *auxin efflux carrier 4* (*PIN4*), *endo-1,4-beta-glucanase* (*ENG*), pectate lyase like (PLL), and *Formin 2A* [33–36]. These giant cell marker genes exhibited high and distinct expression patterns in clusters 16, 25, and 26. More specifically, the giant cell and cell cycle marker genes *CCS52B*, *cdc2b*, *CycB1;1*, *CycA2;1*, *MAP65–3*, *alpha- and beta-tubulins*, and *Formin 2B* were highly and specifically expressed in ~30% and 50% of the cells in clusters 16 and 26, respectively (Fig. 1d). The giant cell marker genes *Aux1*, *PIN4*, *ENG*, *PLL*, and *Formin 2A* were abundantly and specifically expressed in ~70% of the cells in cluster 25 (Fig. 1d). The expression patterns of these giant cell marker genes suggest that clusters 16 and 26 captured young giant cells, whereas cluster 25 captured fully developed giant cells. While clusters 25 and 26 contained nuclei exclusively from the 10-dpi galls, ~28% of nuclei in cluster 16 originated from NRC, suggesting that giant cells and NRC share a subset of gene expression programs, as recently reported [7]. However, subclustering of cluster 16 revealed that gall-derived and NRC-derived nuclei separated into distinct subclusters (Fig. S2), indicating that, despite some shared transcriptome, these cells represent distinct cellular identities.

Through differential gene expression analysis, we identified statistically significant differentially expressed genes (DEGs) in all clusters, including 252, 467, and 325 genes preferentially expressed in clusters 16, 25, and 26, respectively (Table S4). After eliminating common genes, a total of 772 genes preferentially expressed in these three clusters were identified and hence were considered as giant cell specifically expressed genes (Table S5). We next conducted enrichment analysis of gene ontology (GO) terms among these genes to determine biological processes associated with giant cell ontogeny. Cluster 16 and 26 specifically expressed genes were similarly enriched in genes involved in mitotic cell cycle and related processes, including microtubule-based processes, spindle organization, phase transition, and regulation of cyclin-dependent protein kinase activity (Fig. S3). Cluster 25 specifically expressed genes were enriched mainly in genes implicated in the regulation of metabolic process, intracellular transport, cell communication, and chromatin remodeling (Fig. S3). Considering the key role of mitotic cell cycle process in giant cell ontogeny [37, 38], it is evident that clusters 16 and 26 contain a higher portion of young giant cells, whereas cluster 25 contains more differentiated giant cells in which metabolic pathways, cellular communication, and vesicle-mediated transport appear to be highly active.

### Validating giant cell-specific gene expression using promoter activity

**Figure 2:**
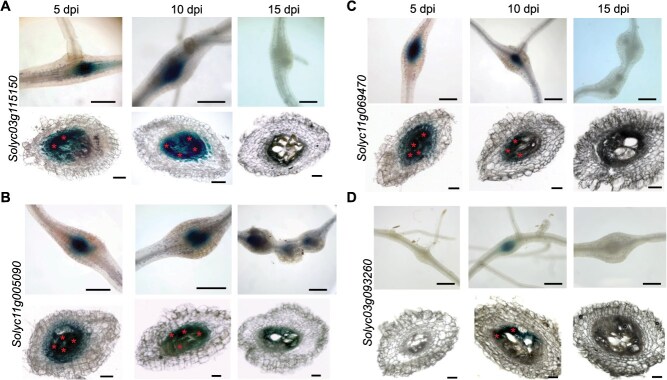
Promoter activity of four giant cell specifically expressed genes following *M. incognita* infection. (a–d) Promoter:GUS constructs for *Solyc03g115150* (**a**), *Solyc11g005090* (b), *Solyc11g069470* (c), and *Solyc03g093260* (d) were generated and expressed in tomato seedlings using transgenic hairy root system. GUS activity was determined in transgenic roots at 5, 10, and 15 days following *M. incognita* infection. The top panel for each gene illustrates GUS activity in galls, while the bottom panel displays GUS activity in giant cells from cross-sections. Giant cells are indicated by asterisks. Scale bars: 350 μm for the top panel and 100 μm for the cross-sectional views in the bottom panels. GUS activity of these promoter constructs in noninfected roots is provided in Fig. S4.

To further validate giant cell-specific gene expression, promoter:GUS (β-glucuronidase) constructs of four genes encoding a histone deacetylase (*Solyc03g115150*,) a cyclin A1 (*Solyc11g005090*), a homeobox leucine-zipper transcription factor (*Solyc11g069470*), and a structural maintenance of chromosomes protein (*Solyc03g093260*) were generated and expressed in tomato seedlings using transgenic hairy root system. After *M. incognita* inoculation, GUS activity was determined at 5, 10, and 15 dpi in both infected and noninfected roots. Noninfected roots did not show any GUS activity at these time points (Fig. S4). Infected roots expressing *Solyc03g115150* promoter construct showed strong GUS staining in galls and giant cells at 5 and 10 dpi but not at 15 dpi (Fig. 2a). *Solyc11g005090* promoter drove strong GUS activity in galls and giant cells at all time points, though weaker at 15 dpi (Fig. 2b). GUS activity driven by *Solyc11g069470* promoter was sporadically detected in galls and giant cells at 5 dpi, became consistently present in all galls and giant cells at 10 dpi, but was completely absent at 15 dpi (Fig. 2c). Promoter activity of *Solyc03g093260* was detected in galls and giant cells only at 10 dpi (Fig. 2d). The promoter activity data strongly support the snRNA-seq results, validating the expression of giant cell-specific genes.

### Developmental trajectories of giant cells

**Figure 3:**
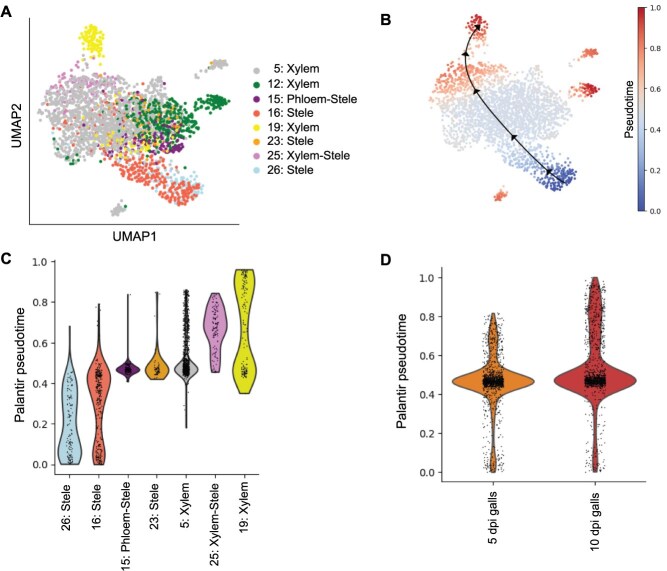
Predicted developmental trajectories of giant cells. (a) Single-cell UMAP plot of eight annotated xylem/stele clusters from galls. Nuclei are colored based on assigned clusters. (b) Pseudotime trajectories of xylem/stele nuclei from cluster 5, 12, 15, 16, 19, 23, 25, and 26 across pseudotime. Cluster 12 was not in the trajectory path. (c) Distribution of xylem/stele nuclei from cluster 5, 15, 16, 19, 23, 25, and 26 across pseudotime showing gradual transition of young giant cells (cluster 26), to intermediate state (cluster 16), to more differentiated giant cells (clusters 5, 25, and 19). Nuclei in clusters 15 and 23 did not show significant changes in gene expression patterns as they move along the pseudotime trajectory. (d) Distribution of xylem/stele nuclei from 5- and 10-dpi galls across pseudotime showing enrichment in early and late pseudotimes, respectively.

snRNA-seq captures the heterogeneity within a cell population and provides a way to examine how cells change as they differentiate, thereby enabling the determination of differentiation trajectories. To predict the developmental progression of giant cells, we generated a UMAP plot of the eight clusters annotated as stele/xylem cell (clusters 5, 12, 15, 16, 19, 23, 25, and 26). Examining the distribution distance of the UMAP plot revealed that cluster 16 was connected to clusters 26 and 25, which were found to contain young and more differentiated giant cells, respectively (Fig. 3a). This topology suggests that cluster 16 contains cells that represent intermediate state of giant cell differentiation. We further examined this suggestion by performing pseudotime analysis through ordering the cells along a trajectory based on their gene expression profiles, which would reflect the developmental progression. Similar to distribution distance of the UMAP plot, the predicted trajectories revealed gradual transition of cells in cluster 26 (young giant cells), to intermediate state (cluster16), to more differentiated giant cells (clusters 5, 25, and 19) (Fig. 3b and c). While cluster 12 was not in the trajectory path, cells in clusters 15 and 23 did not show significant changes in gene expression patterns as they move along pseudotime path (Fig. 3b and c). This may indicate that these cells are not part of the giant cell differentiation process and might be in a more terminal, mature, or quiescent state. We next compared the distribution of cells from the 5 and 10 dpi across pseudotime. Cells from the 5-dpi time point were enriched in early pseudotime, whereas cells from the 10-dpi time point were gradually shifted toward later pseudotime (Fig. 3d). In this context, it may be relevant to mention that clusters 25 and 26 contained nuclei exclusive to the 10-dpi galls, and both clusters were expected to include more differentiated cells. However, our finding that cluster 26 contained young giant cells indicates that both early-stage and more differentiated giant cells were present in the 10-dpi galls. This finding is supported by the distribution of xylem/stele nuclei from 5- and 10-dpi galls across pseudotime (Fig. 3d) and reflects the cellular heterogeneity at this stage of development.

### Giant cell differentiation is mediated through complex gene activation patterns

**Figure 4:**
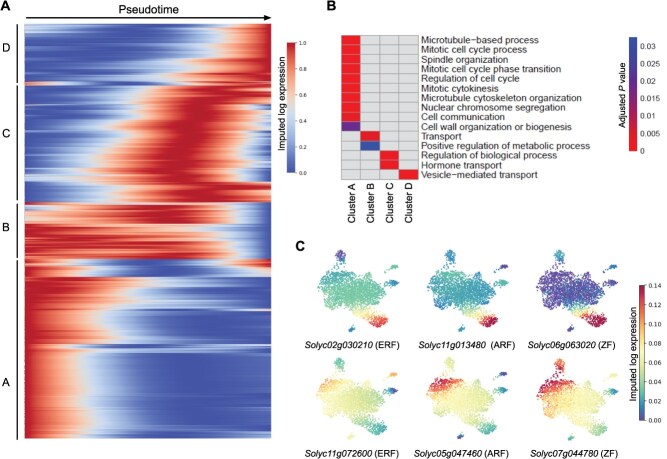
Sorting giant cell specifically expressed genes by pseudotime. (a) Heatmap of 772 giant cell specifically expressed genes, revealing four major gene clusters (A–D) with distinct temporal expression patterns along pseudotime. (b)GO enrichment analysis of genes identified in the four major gene clusters (A–D). The color bar on the right represents the adjusted *P-*value of significantly enriched terms. Analysis was performed using PANTHER version 19.0 with Fisher’s exact test and Bonferroni correction for multiple testing, applying a significance threshold of *P* < 0.05. (c) Expression patterns of six transcription factors of the ERF, ARF, and ZF families across the developmental trajectory of giant cells. Members of the same transcription factor family displayed distinct expression trajectories.

To gain insight into how gene expression changes as giant cells transition through different states, we sorted giant cell specifically expressed genes (772) across pseudotime. Four distinct clusters (A–D) representing distinct temporal expression patterns across pseudotime were observed (Fig. 4a). Cluster A showed high expression during early pseudotime, progressively decreasing across pseudotime, while cluster B exhibited high expression during early and intermediate pseudotime states before similarly decreasing (Fig. 4a). Genes in cluster C and D showed temporal expression patterns specific to intermediate and late pseudotime stages, respectively (Fig. 4a). Genes in cluster A were significantly enriched in mitotic cell cycle and related processes (Fig. 4b), whereas genes in cluster B were enriched in functions related to regulation of metabolic processes and transport (Fig. 4b). Genes in cluster C were enriched in regulation of biological processes and hormone transport. Genes in cluster D were enriched in vesicle-mediated transport (Fig. 4a). Pseudotime progressions of these genes imply that giant cell ontogeny is mediated through complex gene activation patterns.

### Genes associated with specific molecular functions exhibit similar temporal expression patterns across pseudotime

We next examined whether genes associated with specific molecular functions exhibit the same temporal expression patterns across pseudotime. Interestingly, genes encoding the microtubule binding protein TPX2 and B-type cyclins showed high expression during early pseudotime and progressively decreased across pseudotime (Fig. S5a and b). The opposite temporal expression pattern was observed for several genes involved in auxin transport and signaling as well as those involved in polyamine biosynthesis (Fig. S5c and d). These genes showed gradual increases in expression along pseudotime. Several kinesin-like genes displayed high expression during early and/or intermediate pseudotime (Fig. S5e). Genes involved in various aspects of epigenetic modifications, including DNA methylation, histone modifications, and chromatin remodeling showed high expression during early, intermediate, or late pseudotime (Fig. S5f).

### Transcription factor dynamics in giant cells across pseudotime

We further analyzed the expression of transcription factors uniquely expressed in giant cells along pseudotime to gain insights into the regulatory mechanisms driving giant cell differentiation. Notably, a limited number of transcription factors were found to be expressed early in the trajectory (Fig. S5g). These transcription factors belong to the MYB, WRKY, Auxin Response Factor (ARF), AP2-like ethylene-responsive factor (ERF), and zinc finger (ZF) families. As cells progress along pseudotime, more transcription factors were found to be expressed, reflecting a gradual activation of more complex gene regulatory networks as young giant cells adopt specific fates and become more differentiated (Fig. S5g). The growing complexity in transcriptional regulation was mediated mainly through the activity of numerous members of the bHLH, homeobox leucine-zipper, ZF, jumonji, and NAC domain families (Fig. S5g). Notably, members of the same transcription factor families such as ERF, ARF, and ZF exhibited distinct expression patterns across pseudotime (Fig. 4c), suggesting different regulatory roles and functional specialization.

### Silencing giant cell-expressed genes enhanced plant resistance to *M. incognita*

**Figure 5:**
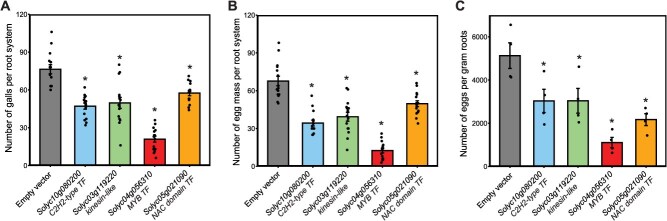
Silencing giant cell specifically expressed genes enhanced plant resistance to *M. incognita**.*** TRV-based VIGS approach was used to silence the expression of four giant cell-specific genes: an MYB transcription factor (*Solyc04g056310*), a C2H2-type ZF transcription factor (*Solyc10g080200*), an NAC domain transcription factor (*Solyc05g021090*), and a kinesin-like protein (*Solyc03g119220*). Plants were inoculated with ~400 second-stage juveniles of *M. incognita*. Six weeks post-inoculation, the number of galls (a), egg masses (b), and eggs per gram of root tissue (c) were quantified. Data are presented as mean ± SE of at least 15 plants, with each dot representing an individual plant. Statistically significant differences between gene-silenced and control plants (empty vector) were determined using ANOVA (*P* < 0.05) and are indicated by asterisks.

We used tobacco rattle virus (TRV)-based virus-induced gene silencing (VIGS) approach to silence four giant cell-expressed genes, encoding an MYB transcription factor (Solyc04g056310), a C2H2-type ZF transcription factors (Solyc10g080200), an NAC domain transcription factor (Solyc05g021090), and a kinesin-like protein (Solyc03g119220) (Table S5). Reverse transcription-quantitative polymerase chain reaction (RT-qPCR) analysis revealed that TRV2 vectors generated to specifically silence these genes achieved between 45% and 60% downregulation in randomly selected root tissues (Fig. S6). All silenced plant lines exhibited significant decreases in susceptibility, as assessed by quantifying the number of galls and egg masses per root system, as well as the number of eggs per gram of root tissues (Fig. 5a–c). Among the tested lines, the MYB transcription factor (*Solyc04g056310*) demonstrated the greatest reduction, with an approximate decrease of 80% (Fig. 5a–c). These findings provide evidence for the functional involvement of these genes in mediating plant susceptibility to *M. incognita*.

### Cell type-specific response to nematode infection

**Figure 6:**
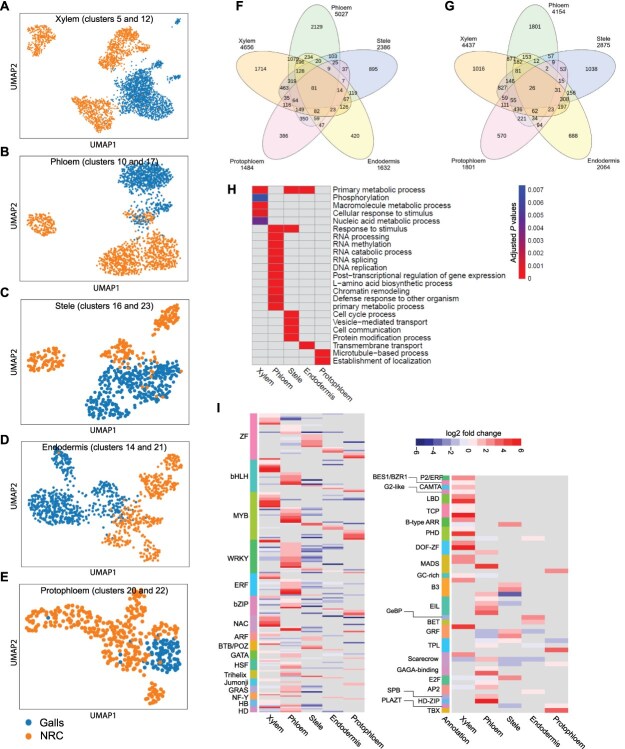
Cell type-specific response to *M. incognita* infection. (a–e) Visualization of gall (blue) and control cells (orange) within xylem (a), phloem (b), stele (c), endodermis (d), and protophloem (e) clusters via UMAP plots. Note that within each of these five cell types, gall and control cells exhibited distinct separation, and therefore used for differential gene expression analysis. (f, g) Venn diagrams showing numbers of unique and overlapping genes among upregulated (f) and downregulated (g) genes identified in xylem, phloem, stele, endodermal, and protophloem cells in response to *M. incognita* infection. Gene lists were provided in Tables S6–S10. (h) GO enrichment analysis of nonoverlapping nematode-responsive genes identified in xylem, phloem, stele, endodermal, and protophloem cells upon *M. incognita* infection. Analysis was conducted as described in Fig. 4. Gene lists were provided in Tables S11–S15. (i) Heatmap displaying nonoverlapping expression profiles of 264 transcription factors across xylem, phloem, stele, endoderm, and protophloem cells. Fold change values of these transcription factors across various cell types were provided in Table S16.

Our analysis revealed that several cell type-specific clusters contained cells from both galls and control samples. Therefore, we reasoned if cells from gall and control samples within each cluster can be distinguishably separated, comparative gene expression analysis can be conducted to accurately determine cell type-specific responses to nematode infection. Two cell type-specific clusters for xylem (5 and 12), phloem (10 and 17), stele (16 and 23), endodermis (14 and 21), and protophloem (20 and 22) containing a high number of gall and control cells were selected for differential gene expression analysis. Notably, within each of these 10 clusters, gall and control cells exhibited distinct separation at each time, though some similarities persisted across time points. Thus, we performed gene expression analysis by merging gall or control cells across both time points and clusters (Fig. 6a–e). A total of 9093, 9181, 5261, 3696, and 3285 genes were identified as differentially expressed in xylem, phloem, stele, endodermal, and protophloem cells, respectively, in response to nematode infection (Tables S6–S10), implying that these cells underwent extensive transcriptome programing in response to nematode infection. After eliminating genes that were similarly up- or downregulated in more than one cell type, 2730, 3930, 1933, 1108, and 956 genes were identified as nematode-specific responsive genes in xylem, phloem, stele, endodermal, and protophloem cells, respectively (Fig. 6f, g and Tables S11–S15). GO term analysis revealed that xylem, phloem, and stele cells were enriched in genes involved in primary metabolic processes and cellular response to stimulus (Fig. 6h). Genes involved in RNA metabolism (processing, methylation, splicing, and degradation), post-transcriptional regulation of gene expression, and chromatin remodeling were specifically enriched in the gall phloem cells (Fig. 6h). Genes involved in metabolic processes of macromolecule and nucleic acid were significantly enriched in xylem cells. Genes related to vesicle-mediated transport and cell communication were enriched in the stele cells. These findings suggest that the diverse cell populations surrounding giant cells are transcriptionally reprogrammed and actively participate in the biological processes and molecular functions necessary to maintain giant cells as functional feeding structures.

To gain insights into gene regulatory programs that govern plant cell type-specific responses to nematode infection, we identified upregulated transcription factors among the nematode-specific responsive genes determined in each of these five cell types. A total of 264 cell type specifically expressed transcription factors were identified with the majority being detected in phloem (104) and xylem (86) cells (Fig. 6i and Table S16). More than half of these regulatory factors belong to the ZF, bZIP, bHLH, MYB, WRKY, and ERF families (Fig. 6i and Table S16), suggesting key roles of these family members in regulating cellular-specific responses to nematode infection. Interestingly, members of certain families appeared to exhibit cell type-specific expression in response to nematode infection. For example, all members of the TCP were specifically upregulated in xylem cells, whereas members of the GAGA-binding, GRAS, and EIN3 families were specifically expressed in phloem cells (Fig. 6i). Members of large gene families such as WRKY and ERF exhibited preferential expression in phloem cells. Single members of the GeBP and BET families were uniquely expressed in endodermal cells (Fig. 6i). Similarly, single members of the PLATZ and TBX were exclusively expressed in protophloem cells in response to nematode infection (Fig. 6i).

## Discussion

In this study, we show that snRNA-seq provides an unparalleled toolset for investigating the transcriptome of galls induced by *M. incognita* on tomato roots by offering high-sensitivity, cell-specific resolution, and the ability to predict the developmental trajectories of giant cells. Using giant cell marker genes, we identified three stele clusters (clusters 16, 25, and 26) that captured young and more differentiated giant cells. Gene expression analysis revealed that 772 genes were specifically expressed in these clusters, designating them as giant cell specifically expressed genes. Notably, several genes encoding cyclins, kinesin-like proteins, and TPX2 were the most active in clusters 16 and 26. The high activity of these genes reflects the extraordinary requirements for cell cycle regulation, cytoskeletal remodeling, and spindle assembly in young giant cells as they transition into highly specialized and enlarged feeding cells. GO term analysis of specifically expressed genes in clusters 16 and 26, revealed significant enrichment for mitotic cell cycle and related processes. This finding further supports the notion that clusters 16 and 26 captured young giant cells, considering the well-established roles of these cellular processes in giant cell formation [37–39].

Once giant cells transit to mature and fully functional feeding cells, they acquire new functions and specialized characteristics to meet the demands of their role as nutrient sources for parasitic nematodes [8, 39, 40]. Our data provided insights into these characteristics and revealed significant enrichment for genes involved in various metabolic pathways, cellular communication, and vesicle-mediated transport among the specifically expressed genes in cluster 25, which appears to capture more differentiated giant cells. Among this gene list, we found the expression of specific membrane transporters for sugars, polyamines, ions, and nutrients to be highly upregulated, supporting the potential critical roles of these transporters in fulfilling the heightened nutrient demands of giant cells to support nematode development and maturation.

Cellular communication and vesicle-mediated transport appear to play key roles in giant cell function. This is supported by upregulation of several genes governing vesicle formation, trafficking, docking, and fusion such as those coding for exocyst complex components, myosins, vacuolar and vesicle-associated proteins, NSF attachment proteins, reticulon-like protein, alpha-taxilin, ARF guanine-nucleotide exchange factors, clathrin interactors EPSIN 1 and EPSIN 2, and AP-1 complex subunits gamma-1, mu-1, and epsilon, among others [41, 42]. Notably, several genes involved in cellular differentiation and enlargement were specifically expressed in cluster 25, including several expansins, which control cell growth and expansion, homologs of BLISTER, which prevent premature differentiation [43], a regulatory-associated protein of TOR 1, which stimulates cell growth [44, 45], an OVATE family protein, which controls multiple aspects of plant growth and development [46], and a STRUBBELIG-receptor, which controls cell morphogenesis and organ development [47, 48]. This finding suggests that cellular differentiation and growth are tightly controlled in fully developed giant cells to maintain integrity and function over the extend time of nematode feeding.

Our results suggest that pseudotime analysis accurately captured the sequential progression of the biological processes associated with giant cell differentiation and development. Consistent with the fundamental roles of mitotic cell cycle and related processes in early stages of giant cell formation [38, 39], genes involved in these processes exhibited high expression during early pseudotime (cluster A in Fig. 4). In contrast, genes involved in cellular processes associated with more differentiated giant cells such as metabolism, vesicle trafficking, and transport [8, 49–52] were highly expressed during late pseudotime (clusters B, C, and D).

Consistent with the role of epigenetic mechanisms in nematode parasitism [16], genes involved in various aspects of epigenetic modifications showed peak activity during early, intermediate, or late pseudotime, suggesting their involvement in regulating giant cell differentiation and phase transition. Gene annotations suggest that DNA hypomethylation may facilitate the priming of infected cells for differentiation, while histone deacetylation drives transcriptional changes essential for differentiation, and histone methylation, along with chromatin remodeling, contributes to stabilizing the differentiated state. Cell fate trajectories analysis also revealed that giant cell differentiation is regulated by a dynamic activation of transcription factors, with early-stage factors from the MYB, WRKY, ARF, and ERF families, giving way to increasing complexity involving more regulators from the bHLH, homeobox, ZF, and NAC domain families as differentiation progresses. Our data showing that silencing members of the MYB, ZF, and NAC domain families significantly increased plant resistance (Fig. 5) further support their crucial roles in promoting plant susceptibility. It would be interesting to determine the functional redundancy or specificity of these factors and how interactions among them regulate giant cell differentiation and transition from early stage to late stage of development.

The transcriptomes of various cell types surrounding giant cells altered substantially with approximately two-thirds of DEGs being shared between at least two cell types (Fig. 6f and g). These shared programs may facilitate coordinated cellular responses, including immune signaling, structural support, and metabolic adjustments, which are critical for supporting giant cell functions. Similarly, various cell types of germinating seeds have been shown to exhibit shared transcriptional programs [27]. The specific transcriptional programs of xylem, phloem, stele, endodermal, and protophloem cells surrounding giant cells involve a total of 9557 genes, and support specialized functions such as metabolic processes of macromolecule, RNA processing, post-transcriptional gene regulation, vesicle-mediated transport, and defense responses, all of which play critical roles in establishing the compatibility of plant–nematode interactions [39, 52–55]. A set of 264 cell type specifically activated transcription factors were predicted to regulate cell type-specific gene expression with members of the ZF, bZIP, bHLH, and MYB, WRKY, and ERF families appeared to play key regulatory roles in this specificity. Members of these families were recently reported to drive cell type-specific functions [24, 56, 57].

Interestingly, our snRNA-seq analysis detected roughly one-third and half of DEGs identified by bulk RNA-seq experiments using tomato whole-infected roots [6] or gall tissues [7], respectively. This high overlap not only demonstrates the technical robustness of our nuclei-isolation and library-preparation workflow but also confirms that nuclear transcriptomes faithfully reflect key biological signals. By capturing such a broad set of gene expression changes, snRNA-seq substantially expands our capacity to detect distinct and even rare cell states, an advantage that becomes critical when whole giant cell isolation is technically challenging or simply infeasible.

In conclusion, our analysis resulted in the identification of specifically expressed modules in giant cells, enabling the prediction of their developmental trajectories and the associated biological processes, and hence better understanding of giant cell ontogeny. The identified 772 giant cell specifically expressed genes provide genuine targets for engineering resistance by enhancing plant defenses, disrupting feeding site formation, and preventing successful parasitism. Assessing promoter activity of a few of these genes pointed to specific inducibility in giant cells (Fig. 2). Discovering highly specific nematode-inducible promoters may guide future engineering of resistant tomato cultivars. For example, these promoters would enable targeted activation of immunity genes upon nematode infection, offering an innovative solution to a persistent agricultural problem. Additionally, a large set of genes preferentially expressed in specific cell types of nematode-induced galls were identified, providing a valuable resource for refining models of tissue organization and cellular specialization.

## Materials and methods

### Plant materials and growth condition

All experiments described in this study were conducted using the nematode-susceptible tomato (*Solanum lycopersicum*) cultivar Heinz 1706. The plants were cultivated in controlled growth chambers maintained at 26°C with a 16-h light and 8-h dark photoperiod, and 65% humidity.

### Nematode inoculation and sample collection

Nematode inoculation and tissue collection were performed as previously described [7]*.* In brief, 2-week-old tomato seedlings, growing in a sterilized mixture of sand and topsoil, were inoculated with ~250 second-stage juveniles of *M. incognita* per seedling. Inoculated seedlings were maintained at the same growth conditions described above*.* Galls, at the swellings ends, and NRC were dissected and collected at 5 and 10 dpi. At each time point, ~500 galls and an equal number of neighboring regions were collected from at least 96 plants, ensuring robust coverage of biological variability. Collected tissues were used for nuclei isolation and snRNA-seq library construction.

### Nuclei isolation, snRNA-seq library construction, and sequencing

Frozen tissue samples from tomato galls and NRC were transferred to SeqMatic LLC (Fremont, CA, USA) in dry ice for nuclei isolation, library preparation, and sequencing. Frozen tissue was carefully transferred to a gentleMACS M tube that was filled with 5 ml of Honda buffer (2.5% Ficoll 400, 5% Dextran T40, 0.4 M sucrose, 10 mM MgCl2, 1 μM DTT, 0.5% Triton X-100, 1 tablet/50 ml cOmplete Protease Inhibitor Cocktail (Roche), 0.4 U μl − 1, Protector RNase Inhibitor (Sigma-Aldrich), 25 mM Tris–HCl, pH 7.4). This buffer composition enables efficient lysis of cell membranes while keeping the nuclear membranes intact. The M tubes were placed onto a gentleMACS Dissociator, where a specific program was executed at 4°C to disrupt the tissue and release the nuclei. The resulting suspension was filtered through a 70-μm strainer and centrifuged at 700 g for 10 min at 4°C. Finally, the pellet was resuspended carefully in 500 μl Honda buffer and filtered through a 30-μm strainer. Nuclei suspensions were loaded on the Chromium X with a targeted cell recovery of 10 000 nuclei. The 30-μm strainers were washed with Honda buffer to determine whether large nuclei from giant cells had been trapped and excluded during filtration. No nuclei were detected in the washing buffer, indicating that the filtration process retained nuclei from all cell types, including giant cells.

snRNA-seq libraries were generated using the Chromium Next GEM Single Cell 3′ Kit v3.1 following the manufacturer protocol (Manual part CG000315, Rev E; 10x Genomics). Library quality was validated using an Agilent TapeStation 4200 (High Sensitivity D1000 ScreenTape to evaluate library composition and size). Preliminary spike-in sequencing was performed, and samples were pooled to equal read counts. Final library concentration was confirmed using qPCR on a Roche LightCycler 96.

The constructed libraries were sequenced on the NovaSeq X Plus with the following read lengths: read 1: 151 bp, index 1: 10 bp, index 2: 10 bp, read 2: 151 bp. Base masking was used to trim all samples to 28 bp in read 1 and 90 bp in read 2. The constructed libraries were sequenced on one lane of a NovaSeq X 10B 300 flow cell.

### Preprocessing of raw snRNA-seq data

The raw snRNA-seq data was analyzed using Cell Ranger 7.2.0 (https://www.10xgenomics.com/support/software/cell-ranger/downloads). Tomato (*S. lycopersicum*) reference genome version (SL3.0) and the annotation files of were downloaded from the Ensemble Plants database (http://plants.ensembl.org/Solanum_lycopersicum/Info/Index). The analysis achieved an average mapping efficiency of 82% and a sequencing saturation of 80% (Table S1). For the subsequent analysis, we used the gene–cell matrix files, produced by Cell Ranger, which contains raw transcript counts of each gene for every individual cell in a single-cell RNA sequencing sample.

### Quality control, data integration, clustering, and annotation

Ribosomal transcripts were excluded, and low-quality cells with <200 detected genes, along with genes detected in <20 cells, were filtered out using the Scanpy package [58]. Potential doublets in each snRNA-seq dataset were identified and removed using DoubletFinder (v.2.0.2) [59]. Ambient mRNA was also removed using SoupX (v1.5.0) [60]. The read counts were then normalized using Scran’s pooling-based size factor estimation method [61], and highly deviant genes (genes exhibiting significantly variable expression profiles across cells within a sample) were calculated using the R package scry [62]. Principal component analysis (PCA) analysis was performed using Scanpy package and visualization with nonlinear dimensionality reduction algorithms, t-SNE, and UMAP was conducted subsequently. To perform clustering, data files for each of the four samples were integrated using the HarmonyPy package (https://github.com/slowkow/harmonypy). The UMAP plot was generated, and the Leiden algorithm was used to cluster the cells with the resolution parameter set to 1.5. Previously validated tomato cell type-specific marker genes [63] and conserved orthologous of known Arabidopsis cell type marker genes [64–67] were used to manually allocate each cell cluster to a specific cell type. Tomato orthologous of known Arabidopsis cell type marker genes were identified using the Ensembl Biomart Plant database (https://plants.ensembl.org/info/data/biomart/index.html). All cell type marker genes used to annotate the clusters are provided in Table S2.

Cluster-specific DEGs for each cluster were identified using scanpy.tl.filter_rank_genes_groups function (min_in_group_fraction and max_out_group_fraction were set to 0.2). GO enrichment analysis for cluster specific DEGs in each cluster were conducted using PANTHER (version 19.0) with corrected *P*-values <0.01 for significance.

### Pseudotime analysis

To predict the developmental trajectories of giant cells, we conducted Palantir pseudotime analysis using the five clusters belonging to the stele/xylem cell populations (clusters 15, 16, 23, 25, and 26). Cells belonging to the control treatments were excluded from these clusters. Preprocessing steps such as normalization, selection of highly variable genes, and PCA were conducted on the raw counts obtained from the previous step, as described earlier. After data preprocessing, diffusion maps analysis and MAGIC imputation were performed using Palantir package (v.1.3.3) [68]. Based on the G1 cell cycle marker genes [7] and the appropriate coordinate position in t-SNE map, a start- and a terminal-state cell were selected. Then, cells were organized along pseudotime and branch probabilities were calculated using Palantir package. The visualization of gene expression along pseudotime on UMAP was performed using the Scanpy package. To illustrate the trajectories, the UMAP coordinates of cells specific to each branch across pseudotime were interpolated and plotted using the plot_trajectory function from the Palantir package.

To determine gene expression patterns of giant cell specifically expressed genes along the pseudotime, a dataframe of gene trends for the 772 genes was extracted. These data were then used to generate a clustermap with the Seaborn package [69], applying hierarchical clustering using the complete linkage method and Euclidean distance metric. Expression patterns of genes associated with certain molecular functions along different branches were visualized on heatmaps using the plot_gene_trend_heatmaps function from the Palantir package. Generation of other plots were conducted using various R packages, such as pheatmap, ggplot2, and RColorBrewer [70–72].

### Identify cell type-specific differentially expressed genes in response to nematode infection

Two cell type-specific clusters for xylem (5 and 12), phloem (10 and 17), stele (13 and 23), endodermis (14 and 21), and protophloem (0 and 22) were selected to identify DEGs in response to nematode infection in a cell type-specific manner. These 10 clusters were selected because they contained a high number of cells from both gall and control samples. For each of these five cell types, raw counts from both time points and both clusters were combined and then divided into three replicates using the NumPy package as previously described [73, 74]. Differential gene expression analysis was performed for each cell type using the pyDESeq2 package [75] by comparing xylem, phloem, stele, endodermal, and protophloem cells from galls with their corresponding cell types in control samples within each cluster. Functional annotations of DEGs were conducted using the tomato gene model (ITAG3.0) from the Solgenomics database (https://solgenomics.net/ftp/tomato_genome/).

### Promoter activity in galls and giant cells using transgenic hairy roots

The promoter sequences of *Solyc03g115150*, *Solyc11g005090*, *Solyc11g069470*, and *Solyc03g093260* (~2000 bp upstream of the translation start codon ATG) were amplified from the genomic DNA of the tomato cultivar Heinz using PCR. The amplified fragments were then cloned upstream of the GUS gene in a binary vector using restriction enzymes. The binary vector contains green fluorescent protein (GFP)-selectable marker to facilitate the identification of transgenic hairy roots. The binary plasmids were confirmed by sequencing and then transformed into *Agrobacterium rhizogenes* strain K599 and used to generate composite plants with transgenic hairy roots as previously described [7]. Composite plants with GFP-positive roots were then inoculated with ~400 second-stage juveniles of *M. incognita*. Histochemical GUS activity of inoculated and noninoculated roots was determined at 5, 10, and 15 dpi.

### Genes silencing using tobacco rattle virus-based VIGS

The TRV-derived vectors TRV1 and TRV2 were used to silence four DEGs (*Solyc04g056310*, *Solyc10g080200*, *Solyc05g021090*, and *Solyc03g119220*) following the method recently described [76]. About 300 bp highly specific to each gene was synthesized and inserted into the pTRV2 vector at multiple cloning sites using restriction enzymes. The pTRV2 vectors with target gene fragments and the pTRV1 vector were separately transformed into *Agrobacterium tumefaciens* strain LBA4404 using the heat shock method. The *Agrobacterium* cultures were grown at 28°C in the dark for 16 h, adjusted to an optical density of 1.0, and suspended in an inoculation buffer containing 150 μM acetosyringone. Cultures containing pTRV1 and the constructed pTRV2 plasmids were mixed at a 1:1 ratio and used to vacuum-infiltrate the roots of 7-day-old tomato seedlings (cv. Heinz 1706). *Agrobacterium* cultures containing pTRV1 and the empty pTRV2 vectors were used as a control treatment. The seedlings were vacuum-infiltrated three times each for 90 s. After 14 days, gene silencing levels were assessed using RT-qPCR, and the seedlings were planted in a mixture of sterilized sand and soil, and then inoculated with ~400 second-stage *M. incognita* juveniles per seedling. Five weeks after nematode inoculation, the number of galls, egg masses, and eggs per gram of root tissues was counted. Statistically significant differences between silenced and control plants were determined using analysis of variance (ANOVA) (*P* < 0.05).

## Supplementary Material

Web_Material_uhaf223

## Data Availability

snRNA-seq data were submitted to the NCBI Gene Expression Omnibus (GEO) database under the accession number GSE289841.
