# Supplementary material for: Single-nucleus RNA-sequencing reveals the cellular programs driving nematode-induced giant cell formation in tomato
Source: Hortic Res. 2025 Aug 22;12(11):uhaf223. doi: 10.1093/hr/uhaf223 (PMC12596086; doi:10.1093/hr/uhaf223)
Supplement: Web_Material_uhaf223 [file web_material_uhaf223.zip › Supplementary Figure 1.pdf]

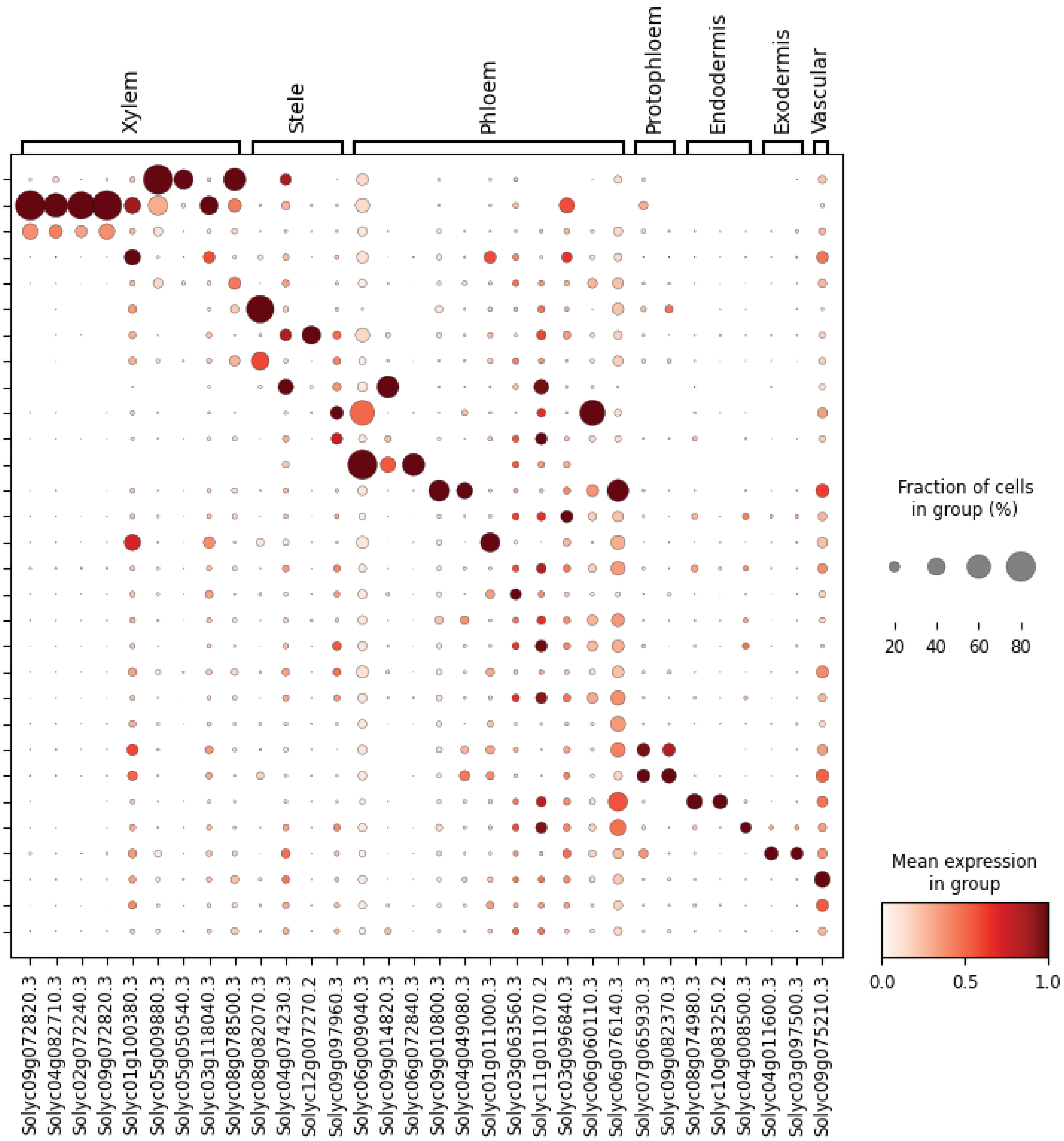

Supplementary Figure 1: Dot plot showing expression of top marker genes for various cell types across all clusters.

Dot diameter indicates the percentage of cells expressing a given marker gene in the indicated cluster. The color bar on the right indicates relative gene expression. The full list of cell-type marker genes is provided in Supplementary Table 2.
