# Supplementary material for: Single-nucleus RNA-sequencing reveals the cellular programs driving nematode-induced giant cell formation in tomato
Source: Hortic Res. 2025 Aug 22;12(11):uhaf223. doi: 10.1093/hr/uhaf223 (PMC12596086; doi:10.1093/hr/uhaf223)
Supplement: Web_Material_uhaf223 [file web_material_uhaf223.zip › Supplementary Figure 2.pdf]

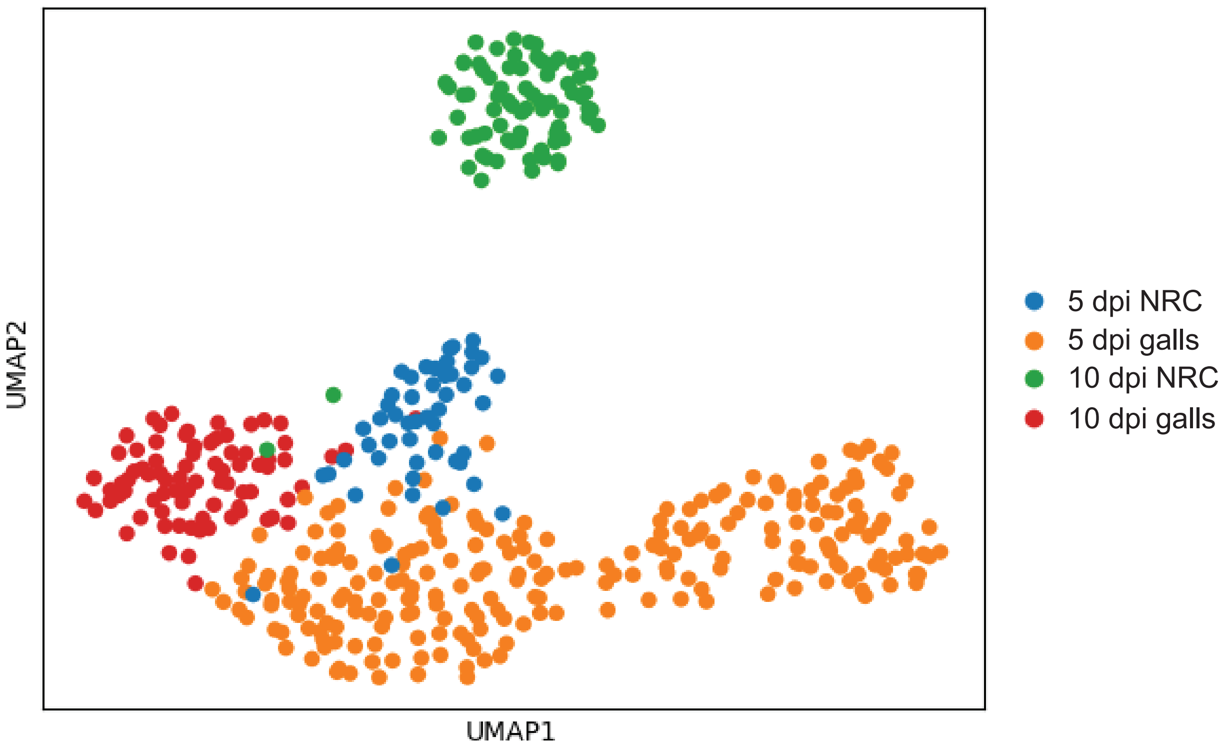

Supplementary Figure 2: Subclustering of cluster 16 showing that gall-derived and NRC-derived nuclei are separated into four distinct subclusters.

Subclustering was performed using Leiden clustering based on gene expression profiles.
