# Supplementary material for: Single-nucleus RNA-sequencing reveals the cellular programs driving nematode-induced giant cell formation in tomato
Source: Hortic Res. 2025 Aug 22;12(11):uhaf223. doi: 10.1093/hr/uhaf223 (PMC12596086; doi:10.1093/hr/uhaf223)
Supplement: Web_Material_uhaf223 [file web_material_uhaf223.zip › Supplementary Figure 3.pdf]

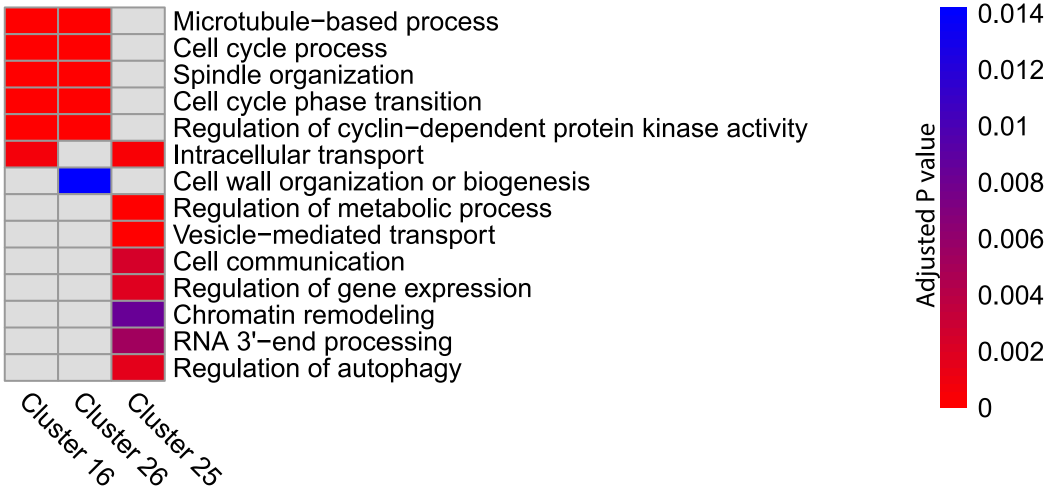


**Supplementary Figure 3: Gene Ontology (GO) enrichment analysis of genes uniquely expressed in clusters 16, 25, and 26.**

The color bar on the right represents the adjusted P value of significantly enriched terms. GO term enrichment analysis was conducted using PANTHER version 19.0, applying Fisher's exact test with Bonferroni correction for multiple testing. A significance threshold of $P < 0.01$ was used.
