# Supplementary material for: Single-nucleus RNA-sequencing reveals the cellular programs driving nematode-induced giant cell formation in tomato
Source: Hortic Res. 2025 Aug 22;12(11):uhaf223. doi: 10.1093/hr/uhaf223 (PMC12596086; doi:10.1093/hr/uhaf223)
Supplement: Web_Material_uhaf223 [file web_material_uhaf223.zip › Supplementary Figure 4.pdf]

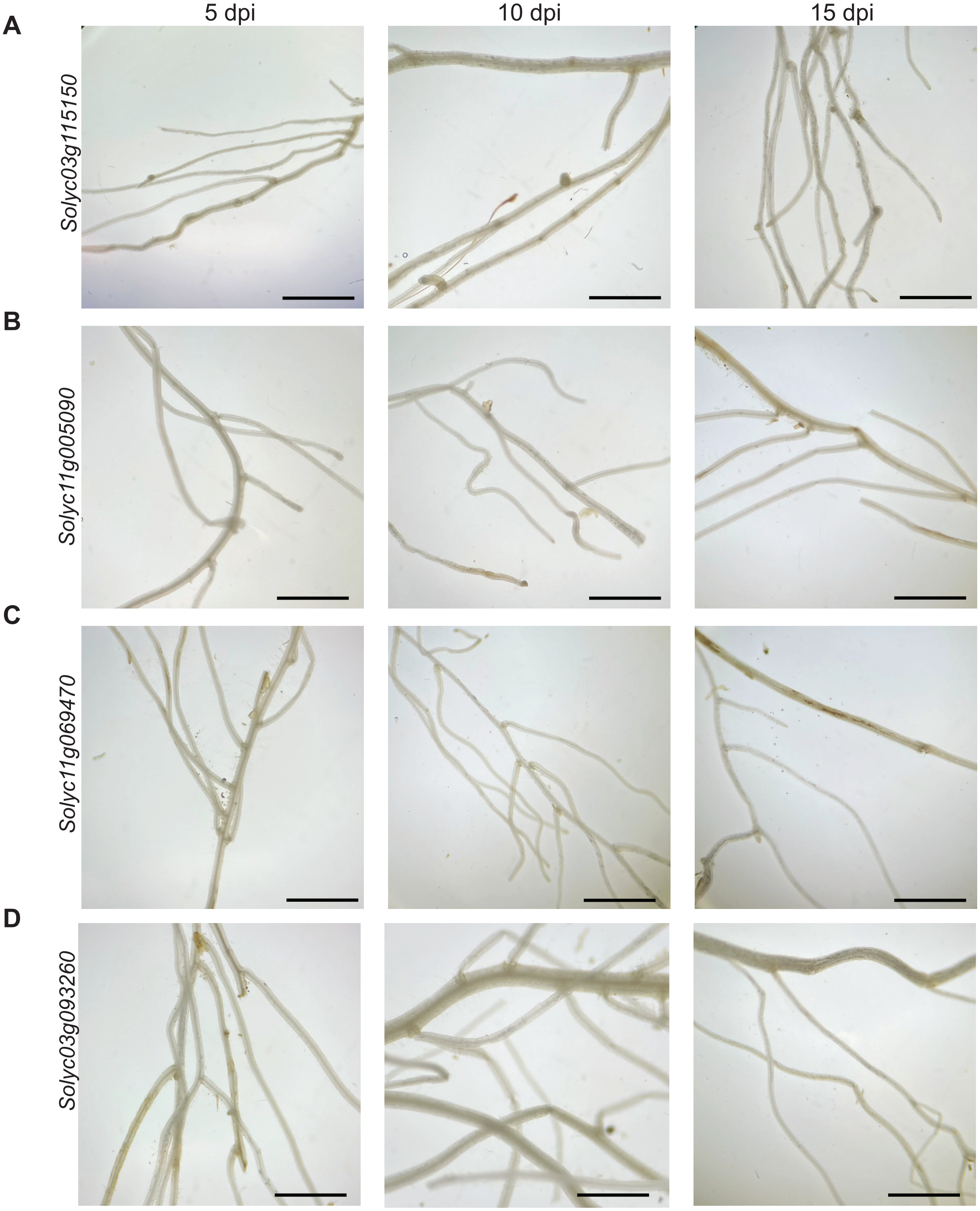

Supplementary Figure 4: Promoter activity of four giant cell-specifically expressed genes in transgenic roots under non-infected conditions.

Transgenic hairy roots expressing promoter:GUS constructs for *Solyc03g115150* (**A**), *Solyc11g005090* (**B**), *Solyc11g069470* (**C**), and *Solyc03g093260* (**D**) were identified using the GFP reporter gene and then subjected to GUS activity. No GUS staining was detected after 16 hours of incubation at 37 ºC. Scale bars = 700 µm.
