# Supplementary material for: Single-nucleus RNA-sequencing reveals the cellular programs driving nematode-induced giant cell formation in tomato
Source: Hortic Res. 2025 Aug 22;12(11):uhaf223. doi: 10.1093/hr/uhaf223 (PMC12596086; doi:10.1093/hr/uhaf223)
Supplement: Web_Material_uhaf223 [file web_material_uhaf223.zip › Supplementary Figure 5.pdf]

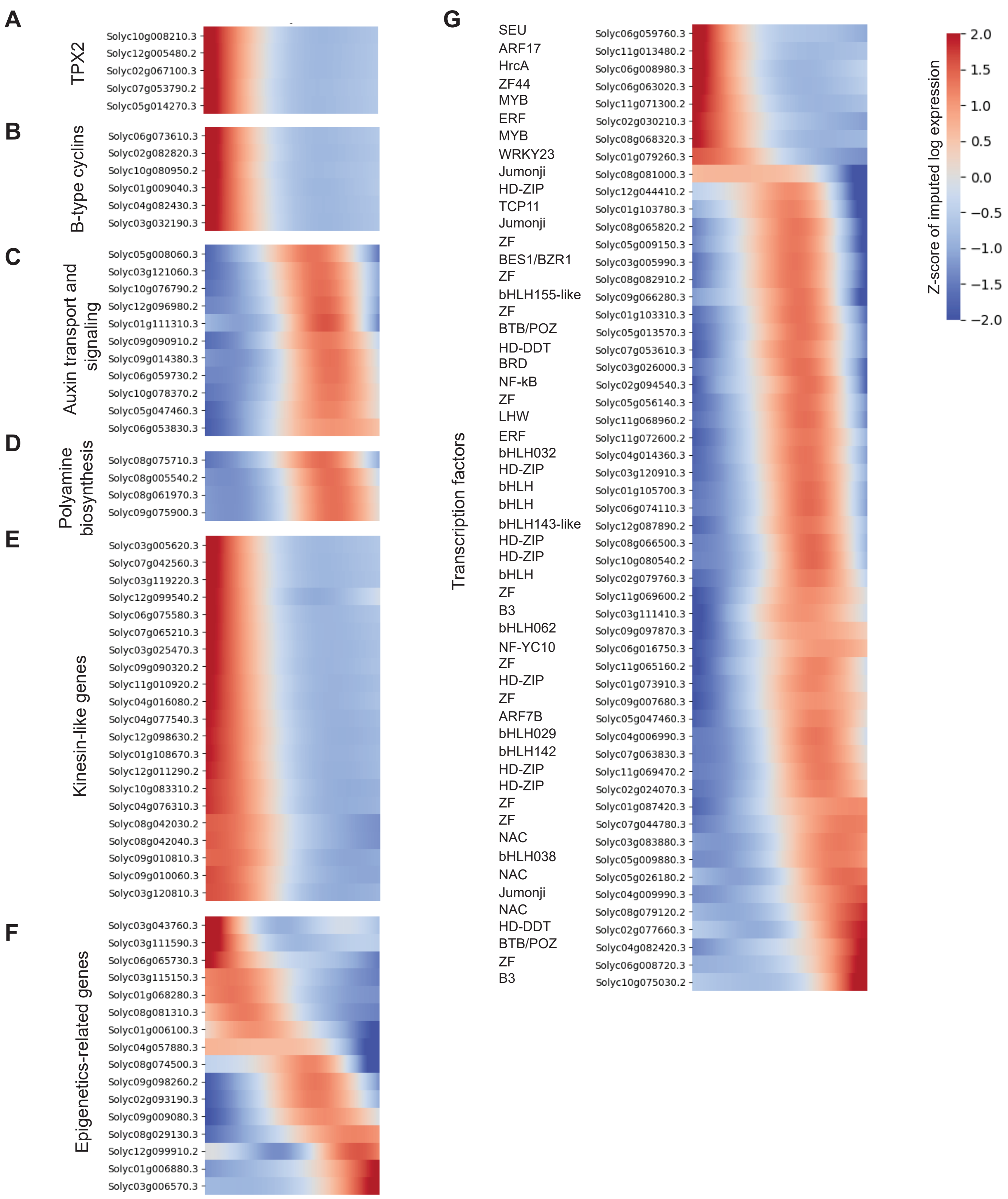


**Supplementary Figure 5: Heatmap illustrating the expression profiles of various giant cell-specifically expressed genes along pseudotime.**

**A**-**G**, Expression patterns of genes encoding TPX2 (**A**), B-type cyclins (**B**), auxin transport and signaling components (**C**), polyamine biosynthesis enzymes (**D**), kinesin-like proteins (**E**), epigenetic regulators (**F**), and transcription factors (**G**) across pseudotime.
