# Supplementary material for: Single-nucleus RNA-sequencing reveals the cellular programs driving nematode-induced giant cell formation in tomato
Source: Hortic Res. 2025 Aug 22;12(11):uhaf223. doi: 10.1093/hr/uhaf223 (PMC12596086; doi:10.1093/hr/uhaf223)
Supplement: Web_Material_uhaf223 [file web_material_uhaf223.zip › Supplementary Figure 6.pdf]

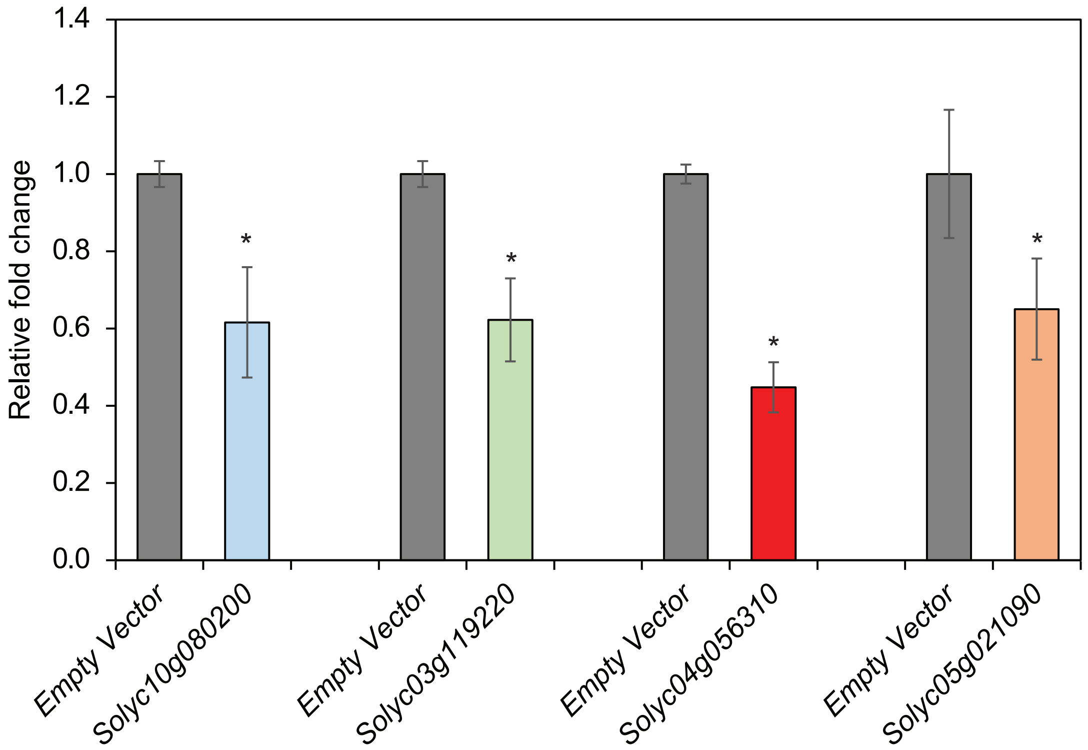

Supplementary Figure 6: RT-qPCR analysis showing significant downregulation of *Solyc10g080200*, *Solyc03g119220*, *Solyc04g056310*, and *Solyc05g021090* in tomato plants (cv. Heinz 1706).

Root tissues were collected at 12 days post-infiltration with *Agrobacterium* suspension cultures carrying pTRV1 and pTRV2 vectors targeting the respective genes. Bars represent the mean ± standard error (SE) of three biological replicates, each with three technical replicates. Statistically significant differences between gene-silenced and control plants (infiltrated with pTRV1 and empty pTRV2 vectors) were determined using ANOVA ($P < 0.05$) and are indicated by asterisks.
